# Image Analysis of Surface Porosity Mortar Containing Processed Spent Bleaching Earth

**DOI:** 10.3390/ma14071658

**Published:** 2021-03-28

**Authors:** Beng Wei Chong, Rokiah Othman, Ramadhansyah Putra Jaya, Doh Shu Ing, Xiaofeng Li, Mohd Haziman Wan Ibrahim, Mohd Mustafa Al Bakri Abdullah, Andrei Victor Sandu, Bartosz Płoszaj, Janusz Szmidla, Tomasz Stachowiak

**Affiliations:** 1 Faculty of Civil Engineering Technology, Universiti Malaysia Pahang, Gambang, Kuantan 26300, Malaysia; bengwei.chong@aiesec.net (B.W.C.); rokiah@ump.edu.my (R.O.); 2 Department of Civil Engineering, College of Engineering, Universiti Malaysia Pahang, Gambang, Kuantan 26300, Malaysia; dohsi@ump.edu.my (D.S.I.); mr.leexiaofeng2018@gmail.com (X.L.); 3 Faculty of Civil and Environmental Engineering, Universiti Tun Hussein Onn Malaysia, Batu Pahat, Johor Bahru 86400, Malaysia; haziman@uthm.edu.my; 4 Center of Excellence Geopolymer and Green Technology, Universiti Malaysia Perlis, Kangar, Perlis 01000, Malaysia; mustafa_albakri@unimap.edu.my (M.M.A.B.A.); sav@tuiasi.ro (A.V.S.); 5 Faculty of Material Science and Engineering, Gheorghe Asachi Technical University of Iasi, 41 D. Mangeron St., 700050 Iasi, Romania; 6 National Institute for Research and Development for Environmental Protection INCDPM, 294 SplaiulIndependentei, 060031 Bucharest, Romania; 7 Department of Physics, Częstochowa University of Technology, 42-214 Częstochowa, Poland; bartosz.ploszaj@pcz.pl; 8 Faculty of Mechanical Engineering and Computer Science, Częstochowa University of Technology, 42-214 Częstochowa, Poland; j.szmidla@imipkm.pcz.pl (J.S.); stachowiak@ipp.pcz.pl (T.S.)

**Keywords:** image analysis, mortar, compressive strength, water absorption, porosity

## Abstract

Image analysis techniques are gaining popularity in the studies of civil engineering materials. However, the current established image analysis methods often require advanced machinery and strict image acquisition procedures which may be challenging in actual construction practices. In this study, we develop a simplified image analysis technique that uses images with only a digital camera and does not have a strict image acquisition regime. Mortar with 10%, 20%, 30%, and 40% pozzolanic material as cement replacement are prepared for the study. The properties of mortar are evaluated with flow table test, compressive strength test, water absorption test, and surface porosity based on the proposed image analysis technique. The experimental results show that mortar specimens with 20% processed spent bleaching earth (PSBE) achieve the highest 28-day compressive strength and lowest water absorption. The quantified image analysis results show accurate representation of mortar quality with 20% PSBE mortar having the lowest porosity. The regression analysis found strong correlations between all experimental data and the compressive strength. Hence, the developed technique is verified to be feasible as supplementary mortar properties for the study of mortar with pozzolanic material.

## 1. Introduction

In recent years, the use of image analysis techniques in civil engineering studies has been increasing, due to their myriad of advantages. Image analysis is a non-destructive test that does not disturb the specimen and can be used for detailed study of the microstructure in new materials such as fiber-reinforced concrete [1]. The primary methodology of image analysis centres around the technology of computed tomography (CT), which is based on absorbing X-ray to visualise the internal microstructure of an object [2]. Other applications of image analysis techniques have been applied to access concrete surface quality and concrete crack under loading [3,4].

Pavani et al. [5] used synchrotron micro tomography to investigate the porosity and pore distribution of concrete subjected to elevated temperatures and managed to put together detailed three-dimensional (3D) models of a specimen along with the diameter and area of the pores. Synchrotron-based X-ray computed microtomography (XRCT) was also used by Promentilla et al. [6] who put together 3D models to investigate concrete deterioration. In another application, the distribution of fiber in fiber-reinforced concrete was studied using a high-caliber CT scanner [7]. However, Ruan and Poursaee [8] experimented with multiple methods to obtain the distribution of fiber in fiber-reinforced concrete and concluded that electrical conductivity measurements were cheaper and easier for obtaining the desired result as compared with advanced CT scanning. Then, Lee et al. [9] concluded in their experiment that the accuracy of the measurement was affected by the diameter of fiber and the image pixel which empathized the need for obtaining high resolution images. This highlights a challenge of image analysis where advanced equipment is often required and may not be practical at a site, in a small-scale laboratory, or under economic constraints. On the plus side, image analysis of concrete surface or crack detection using less sophisticated equipment have also been developed. Szelag [10] studied the cracking pattern of mortar beam using an optical scanner under the resolution of 2400 dpi and eight-bit greyscale. In another experiment, San and Aung [11] captured plastic shrinkage cracks of concrete for image analysis with a mobile mini magnifier. A hand handled microscope has also been successfully utilised for concrete crack analysis [12]. Valikhani et al. [13] evaluated the surface roughness and compressive strength of concrete through images of concrete cross-section. The experiment captured images with only a smartphone camera with 12 megapixels but achieved satisfactory results with the aid of machine learning techniques. By using other advanced computational method such as artificial neural network (ANN), other studies [14,15] have also developed models for concrete strength prediction with images from digital camera. Many studies had developed image analysis techniques to determine the size and distribution of pores. However, each methodology employs different equipment, distances, and procedures, and hence is difficult to replicate or standardise [16].

Thus far, surface porosity or surface bugholes of concrete is mainly considered to be an aesthetic factor which reflects only the surface quality of concrete and mortar [17]. There has been no attempt to correlate the quantified parameter with other measurable material properties. However, some studies have also hinted at the possibility that the surface condition of cementitious products may be a reflection of internal quality, as bugholes are formed due to entrapped air within the material and based on the vibration during the casting process [18]. A recent study by Liu et al. [19] also proved that the mix design of concrete, such as its water/cement ratio and proportion of constituent materials play a role in the formation of surface bugholes. In another study, image analysis of surface bugholes on concrete tunnel lining concluded that surface condition may be affected by workability of the mix, which indirectly correlates this factor with concrete durability [20,21,22,23,24,25,26,27]. Dogan et al. proposed [15] that image processing of concrete could be taken as another non-destructive test similar to ultrasonic pulse velocity and Schmidt hammer to provide additional information for concrete properties prediction. This has led to the belief that the surface condition of concrete and mortar may be established as a factor in the study of its physical properties such as compressive strength.

Furthermore, image analysis of pore and microstructure can be a very potent technique to be applied in the study of mortar and cement with pozzolanic materials as cement replacement. This is because pozzolanic materials improve the performance of mortar and cement through an increase in hydration yield [28], which is reflected by an improved microstructure [29] with less pores [30]. Laboratory assessments of microstructure are commonly performed by conducting water absorption and porosity tests [31], while scanning electron microscope (SEM) is used for the rheological assessment [32]. In this study, we develop a simplified and universal image analysis technique with a simple imaging method that does not require advanced equipment and strict shooting procedure. The image analysis was conducted using ImageJ based software and readily available segmentation plugin. The results are quantified and correlated with other mortar properties from the study of mortar with pozzolanic material as partial cement replacement.

## 2. Materials and Methods

Mortar specimens were prepared using locally available materials. Ordinary Portland cement from YTL Cement, Kuala Lumpur, Malaysia was used. The cement grade was 32.5 N that fulfils the specification of MS EN 197-1: 2014 (2014). The chemical composition and the physical properties of the cement powder are presented on Table 1.

**Figure 1:**
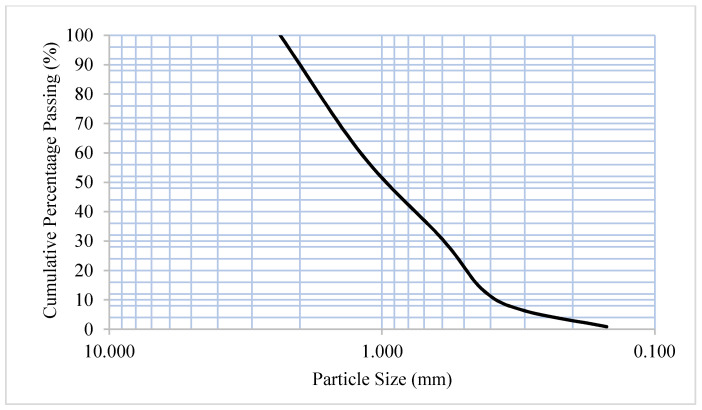
Particle size distribution of fine aggregate.

River sand from YTL Cement (Kuala Lumpur, Malaysia) was used as the fine aggregate to produce mortar. The particle size distribution and grading of the sand was determined through sieve analysis based on BS812: 103. The river sand was sieved through 4.75 mm sieve beforehand, and all particles retained were discarded. For the sieve analysis, the sieves were arranged in descending order, i.e., from 4.75 mm at the top, followed by 3.35, 2.0, 1.18, 0.6, 0.425, 0.300, 0.15 mm, and finally the pan at bottom. Then, 500 g of sand was collected from the batch and placed in the top of the sieve. The top of the sieve was covered by a pan and the sieve shaker ran for 10 to 15 min. During the process, particles with smaller sizes than the sieve passed through, while larger particles were retained. The results are tabulated in Table 2. The particle size distribution plot of river sand was plotted, as shown in Figure 1.

**Figure 2:**
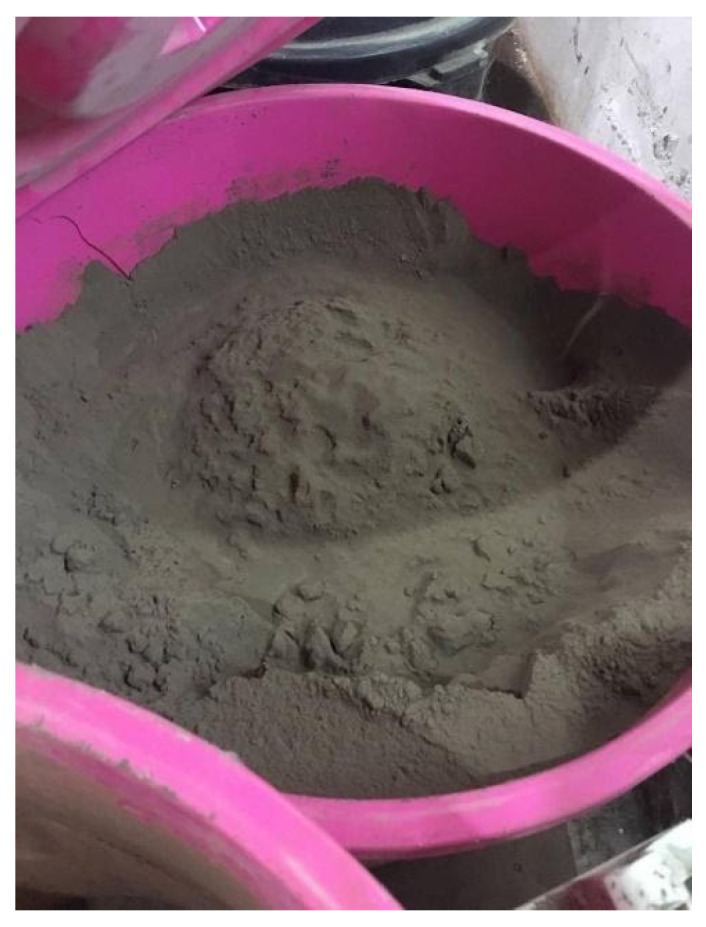
Air-dry processed spent bleaching earth (PSBE).

Processed spent bleaching earth (PSBE) from Kuala Lumpur, Malaysia, which is a pozzolanic material, was used in this study. PSBE is the end product derived from the refining process of palm oil [33]. PSBE was sieved through a 300 μm sieve. The material is shown in Figure 2. The chemical composition of PSBE was analysed and the results are presented in Table 3. According to ASTM C 618-19 (2019), a material can be classified as Class N pozzolan if the combination of silicon dioxide, aluminum oxide, and ferrous oxide is over 70% of its weight. Since the three compounds make up 77.54% of PSBE, it is classified as a Class N pozzolan and can be used as a cement replacement.

## 3. Experimental Work

### 3.1. Mixing Proportion, Casting, and Curing

This study was conducted using type N mortar with varying percentages of PSBE cement replacement. The mix design of mortar was adopted from ASTM C1329-05 (2005), which recommended a cement/sand ratio of 1:2.75. The water/cement ratio was 0.60 for all mixes. Type N mortar was selected as it is most commonly used mortar for constructing exterior and interior load bearing installations, soft stone masonry, and load bearing walls. The minimum target strength of type N mortar is 750 psi or 5.2 MPa. The PSBE replaced cement up to 40% with an interval of 10%. Mortar mixing was carried out under ambient temperature. All required material was measured and placed in a bucket and water was slowly added in three intervals. A heavy-duty mud mixer was used to ensure the uniformity of mixing. Mixing was conducted in three stages with one-third of the water added at each stage and the mixer was run for one minute. Once the mortar formed, the moulds were filled in three layers with a 3 s quick vibration in each filling layer to ensure uniformity of the mix and removal of air pockets while not causing segregation. Demoulding was conducted after 24 h, and the specimens were water cured for up to 28 days.

### 3.2. Flow Table Test

To measure the workability of mortar, a flow table test was conducted, in accordance with ASTM C1437-15 (2015). The apparatus required was from Gilson Company. Inc, Ohio, United States consisted of a round copper table, copper mould, and a meter ruler. Before testing, the round table and mould were thoroughly wetted and cleaned. Then, excess water on the surface was wiped away, and the mould was firmly placed at the middle of the round surface. The mould was filled with fresh mortar using a spade and filling was done in three layers. For every filling, a tamping rod was dropped onto the fresh mortar 20 times. After complete filling, the top surface was struck off using a trowel to obtain a flat surface. The mould was lifted, and the table was raised and dropped with a drop height of 12.5 mm for 25 drops within 15 s. Three readings of mortar diameter were taken at different angles using a meter ruler. The average diameter of mortar was calculated, and the consistency of mortar was determined.

### 3.3. Compressive Strength

A MATEST Autotac 2000kN Automatic Compressive Machine (Treviolo, Italy) was used to carry out the compressive strength test on mortar specimens. The cube mortar specimens for the test had a size of 50 × 50 × 50 mm^3^. The British Standard BS EN 12390-3:2009 (2009) test procedure was adopted. The strength of cube specimens was determined at 1, 7, and 28 days of curing age. Three specimens of mortar cubes from each type of mortar were used for each test to obtain an accurate result.

### 3.4. Water Absorption

The characterization of pore structure in concrete was determined using the water absorption test, in accordance with British Standard BS 1881: Part 122:2011 (2011). The concrete cube specimens required for the water absorption test had a size of 50 × 50 × 50 mm^3^. After 28 days curing, the concrete cubes were dried inside an oven at a temperature of 101 ± 1 °C for 72 ± 2 h. After the drying process, the specimens were cooled in a sealed container for 24 ± 0.5 h. Immediately after the cooling process, the weights of the oven-dry specimens were taken and recorded. Then, the specimens were fully immersed in a water container. To observe the water absorption in close detail, the specimens were weighed at 1, 5, 10, 20, 30, 60, 120, 180, and 1440 min. At each interval, water on the mortar surface was quickly removed by wiping the mortar cubes with a cloth. Then, the mortar specimens were weighed, and the mass was recorded.

### 3.5. Surface Porosity

**Figure 3:**
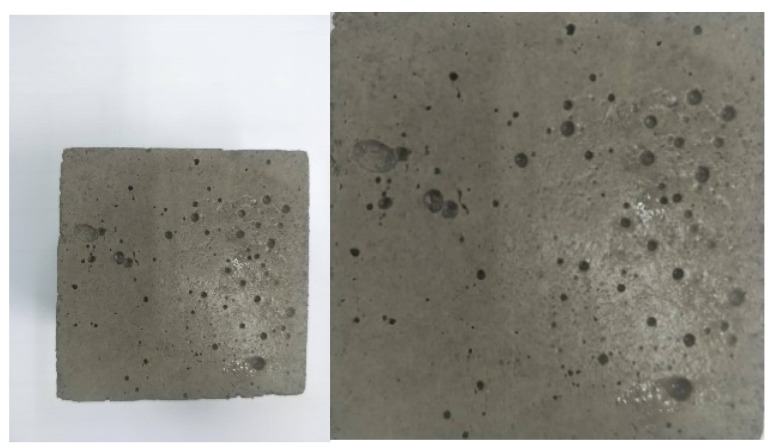
Original and modified images.

The surface porosity of the mortar specimens was investigated using an image analysis technique developed to provide a supplementary quantitative parameter which could be correlated with the mechanical and durability properties of mortar. After 28 days curing, mortar cubes were removed from the curing tank and wiped until the surface was free from moisture. The specimens were placed on a white sheet of paper which served as a background for the imaging process. The camera used has a pixel-per-inch (PPI) of 400. Shooting was done from above with a regular camera at a distant of approximately 30 cm to ensure the entire surface of each mortar specimen fell within the image and included only minimal background space. Curvature of the cube’s edge was made to be not visible in the shooting to provide a two-dimensional (2D) model. Highlight was placed on the centre of the surface to ensure a clear image. For every mortar specimen, all six faces of the cube were shot in a similar manner, resulting in a total of 18 images from three specimens for each mix design. Then, the images were processed to be better analysed. The white background was removed by trimming the photo up to the cement paste to include only the specimen, and the cube was straightened through rotation should it become slanted due to human error. Examples of the original and modified images are shown in Figure 3.

**Figure 4:**
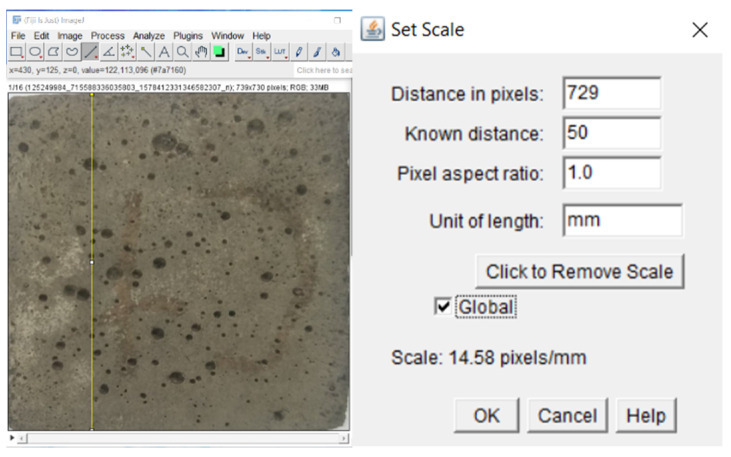
Original and modified images.

Fiji open-source image processing based on ImageJ version 0.8.3 was used to analyse the images. All images were loaded and stacked through scaling every image to that of the smallest size. Scaling was chosen to preserve the measurement and dimension detail; scaling down was chosen as it does not incur a loss in image quality unlike scaling up does. The images were calibrated by taking the straight end-to-end distance of the first image as 50 mm, since the size of the mortar cube was known to be 50 mm. This approach may introduce deviation due to the angle of shooting, but it allows for a global calibration of every image regardless of their original size due to shooting distance, since every image had been scaled to the same size. The error was also minimal because the size of a mortar cube was always the same, and hence an accurate reference. The process of image calibration is shown on Figure 4.

**Figure 5:**
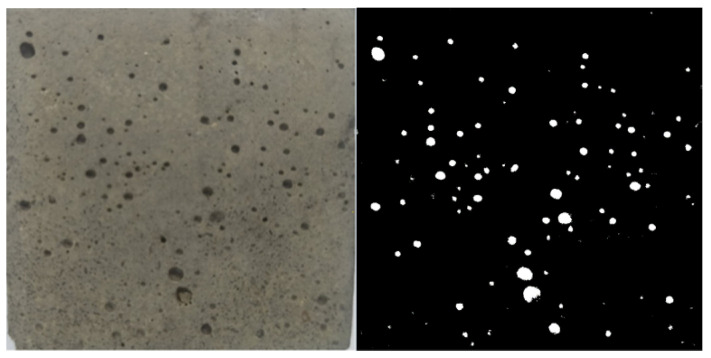
Original image and image thresholding.

Trainable Weka Segmentation published by Free Software Foundation was used to develop a classifier based on two classes, i.e., cement paste and void. The classifier was trained by manual segmentation of a few markings of paste and void on the first five images. Then, the classifier was applied across the entire stack. The researcher went through the pictures and made additional manual segmentation to improve the artificial intelligent in the event of mistakes or inaccuracies. The process was repeated until satisfactory segmentation was found across the stack. The segmentation result was generated. Thresholding was conducted by converting the image into 8-bits and the colour balance of the images was shifted until a black-and-white image of the paste and void remained. The original processed image and the image after thresholding are shown in Figure 5. Finally, Particle analyser is built into Fiji and was conducted to determine the area of the image, the total area of the void, and the percentage of surface porosity of the mortar cubes. The results were analysed to access the viability of the method and the correlation of surface porosity to other mortar properties.

## 4. Results and Discussion

### 4.1. Flow Table Test

Table 4 presents the result of the flow table test. The consistency of control mortar is 155%. It can be deduced that partial replacement of cement with PSBE decreases the consistency of mortar. At 10% and 20% PSBE replacement, the consistency of mortar dropped slightly to 150% and 140%, respectively. However, at 30% and 40% PSBE replacement, a sharper drop in consistency was observed, with the consistency of mortar being 95% and 105%. Pozzolanic materials when used as cement replacement at a fine grind size decreases the workability of cementitious product. The reduction occurs due to the ability of certain pozzolana to absorb water or undergo vigorous reaction during the mixing process [34]. This has been observed in a variety of other pozzolanic materials such as silica fume [35], fly ash [36], and metakaolin [37]. The loss of workability may be minimised or recovered by limiting the percentage of pozzolonic material or through the use of water reducer.

### 4.2. Compressive Strength

Table 5 presents the results of compressive strength for the mortar specimens. The control specimen has a compressive strength of 2.407 MPa at one day; 10 and 20 PSBE specimens have compressive strengths lower than the control specimen, while 30 and 40 PSBE specimens have not developed enough strength to be tested. A low early strength, especially before seven days, is common for cementitious product with pozzolanic material as partial cement replacement [38]. At seven days, the control mortar has a compressive strength of 9.277 N/mm^2^, but 10 and 20 PSBE specimens achieve higher strengths of 10.471 N/mm^2^ and 9.436 N/mm^2^, respectively. Furthermore, mortar specimens with up to 30% PSBE replacement perform better than the control specimen at 28 days. The 20 PSBE specimen has a compressive strength of 12.254 N/mm^2^ after 28 days of curing, which is the highest among all specimens tested in this study. This result agrees with a study on PSBE concrete in which specimens with PSBE replacement had higher compressive strength than a control specimen after 3 days, with the increase in compressive strength becoming more apparent with increased curing age [39]. When the initial hydration process between cement and water has occurred sufficiently, pozzolanic materials such as PSBE react with calcium hydroxide to produce more hydration product [40]. The enhanced secondary hydration fills capillary pores and creates a more durable internal structure, resulting in PSBE mortar that has greater compressive strength than a control. However, an excessive replacement of cement with PSBE can result in a lack of cementitious material in the mix, and hence produces a weaker concrete, as indicated by the 40 PSBE specimen which had a lower strength than the control specimen, despite showing increased strength of pozzolanic reaction with curing age. Hence, the relationship between PSBE and mortar strength is suggested to be curvilinear with the optimal percentage of replacement being 20% for maximum 28-day strength.

### 4.3. Compressive Strength Activity Index

**Figure 6:**
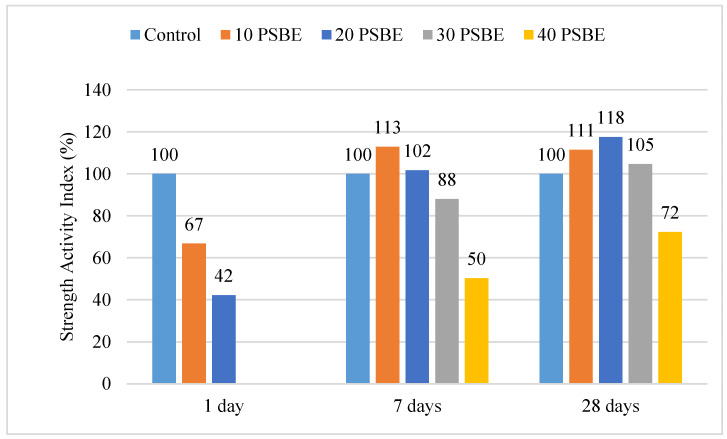
Compressive strength index of eggshell mortar.

Figure 6 presents the strength activity index for the compressive strength of PSBE mortar. According to ASTM C311-18 (2018), the strength activity index is used to determine the strength development of concrete as compared with a control when fly ash or nature pozzolan is used [41]. Since PSBE is a Class N natural pozzolan, the strength activity index can be applied to study the effect of the cement replacement. At the age of one day, 10 and 20 PSBE specimens have a strength indexes of 67% and 42% which are lower as compared with the control. While the data from the 30 and 40 PSBE specimens were not collected, it is clear that PSBE mortar has a poor early strength as compared with regular cement mortar. The strength index of PSBE mortar is low at one day but improves with age because pozzolanic reaction is a long-term process which requires a natural cement hydration process, first, to produce calcium hydroxide (CaOH_2_) [42]. After seven days curing, the strength index of 10 PSBE rises to 113%, which is better than the control specimen. Likewise, 20 PSBE has a strength index of 102%, while the 30 and 40 PSBE specimens achieve 88% and 50% strength indexes as compared with the control specimen. At 28 days, mortar with up to 30% PSBE replacement has a higher strength index than the control concrete and 20 PSBE achieves the highest index which is 118%. At 28 days, sufficient CaOH_2_ is present in the system and PSBE which contains a large among of silica (SiO_2_) begins to combine with calcium hydroxide to produce calcium silicate hydrate (CSH) and contributes towards the long-term strength gain of mortar.

### 4.4. Water Absorption

**Figure 7:**
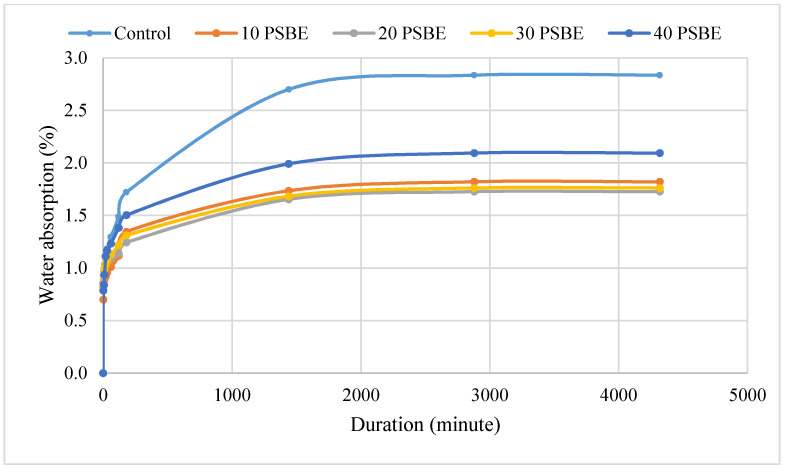
Water absorption of PSBE mortar.

The water absorption of 28-day mortar cubes is plotted on Figure 7. The water absorption rates of all specimens increase in an exponential trend as oven-dry specimens are immersed in water over the course of three days. From the figure, the control specimens have a water absorption rate of 2.70%, which is the highest among all specimens tested. PSBE mortars have greatly reduced water absorption rates of up to 40%; the 40 PSBE specimen has the second highest water absorption, while PSBE replacement of cement at up to 30% shows a significantly reduced water absorption rate which is only 1.65% for the 20 PSBE specimen. Partial replacement of cement with PSBE reduces water absorption rate because the pozzolanic reaction of PSBE produces secondary hydration which increases the amount of hydration product [43]. An increase in hydration product results in smaller internal pores and a denser mortar microstructure which can resist the penetration of water and other aggressive substances [44]. Moreover, fine PSBE particle can serve as a filler of mortar and enhance the space filling properties of the paste.

### 4.5. Surface Porosity

Surface porosity of mortar specimens was obtained by image analysis using Trainable Weka Segmentation and Fiji Particle Analyser. Table 6 shows the output of image analysis for a set of control specimen images. Total area refers to the total area occupied by void on the mortar surface, and average size represents the average size of voids. The percentage area (% area) was the main output of the analysis which showed the percentage of area occupied by void on the concrete surface. A constant value of 255 for the mean refers to the red, green, and blue intensity (RGB) value for white colour of the voids, because a plain black-and-white image thresholding method was adopted. Firstly, the assumed area of a specimen was determined by artificially intelligent to be 2565 mm^2^, calculated by considering the total area and percentage area of the image analysis. This value deviated slightly from the standard area of 50 × 50 mm^2^ mortar cube which was 2500 mm^2^, but the error was minor and within an acceptable range. Hence, the novel methodology employed in this study was feasible. From the dataset below, it can also be observed that the percentage area of surface porosity had a strong fluctuation even for the same concrete mix. As shown in Table 6, the lowest surface porosity was 0.159% (Slice 13) while the highest surface porosity was 4.234% (Slice 14). The fluctuation came from the differences of concrete quality and finishing for every face, as well as the possible influence of workmanship and the condition of the concrete mould. To maximise the reliability of the data, the average porosity of every surface was taken.

Table 7 presents the average surface porosity of mortar specimens. The control specimen had a surface porosity of 2.723%. When PSBE replaced cement, the surface porosity of mortar decreased to 1.664% at 10% PSBE and 1.448% at 20% PSBE. For the 30% PSBE specimen, the surface porosity of mortar increased again to 2.240% but was still lower than the control specimen. For the 40% PSBE specimen, surface porosity was higher than the control. The result was overall positive as it reflected the trend of the water absorption test which was used to similarly measure the microstructure quality of mortar. In this study, 20 PSBE attained the highest 28-day compressive strength among all specimens. The water absorption test reported the lowest water absorption rate for specimens of the same mix, and image analysis of mortar surface further concluded that the 20 PSBE specimen had the least porosity. Hence, the image analysis technique on mortar surface was consistent with other common testing regimes and successfully determined the quality of mortar.

### 4.6. Correlation of Surface Porosity with Mortar Properties

**Figure 8:**
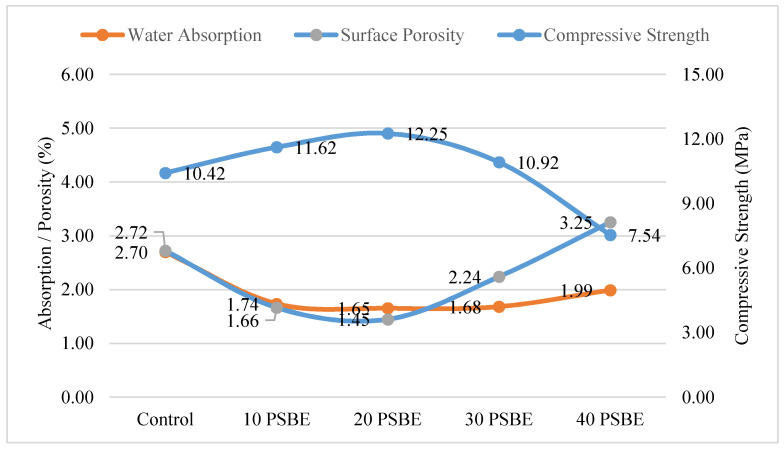
Porosity and compressive strength of mortar.

The reliability of surface porosity as a supplementary parameter of mortar properties was accessed. Figure 8 illustrates the surface porosity, water absorption, PSBE replacement, and mortar 28-day compressive strength on a double-scaled plot. Initial observations indicate that the relationship between mortar strength and percentage of PSBE replacement is curvilinear in nature with an optimal percentage of replacement of 20%. Water absorption of mortar shows an inverse curve with respect to mortar strength, since a lower water absorption rate implies a denser microstructure, and hence higher compressive strength. In addition, the surface porosity data reasonably mimic the trend of water absorption. A regression analysis was performed on all present variables to study the effect of PSBE replacement, water absorption, and surface porosity on the 28-day compressive strength of mortar. The results are tabulated in Table 8. The analysis confirms that the relationship between PSBE replacement and compressive strength is quadratic or curvilinear in nature as presumed during the discussion of mortar compressive strength. The coefficient of determination (R^2^) is 0.9814, which implies that 98.14% of the variance can be explained by the variable. Similarly, water absorption rate of mortar can be correlated with compressive strength in a quadratic function. While the correlation does not imply that the compressive strength of any mortar can always be predicted through water absorption rate, the variable has been used for the prediction of concrete strength [45] and durability [46]. Lastly, the simple linear regression between surface porosity and compressive strength yields a positive result with an R^2^ value of 0.8769, which is identified as a strong correlation (R^2^ > 0.80) between the two variables. While image analysis is only conducted on mortar surface, the high correlation between surface porosity and other factors may indicate that the surface condition of mortar is a reasonable representation of mortar microstructure. The phenomena agree with a similar study which analysed the bugholes of both the surface and internal cross-section of concrete [47]. Hence, the application of image analysis surface porosity is a feasible supplementary parameter to be explored in the study of green cement mortar.

### 4.7. Result Discussion

The image analysis method developed in this study enables the quantification of concrete surface condition into a surface porosity parameter. To date, most image analyses of concrete have been conducted with advanced equipment based on computed tomography (CT) [2,5,6]. CT and X-ray CT provide detailed images of concrete microstructure with graph plot of pore size distribution below 500 µm [5], while, in this study, we evaluated macrostructure of concrete surface based on bugholes with an average size between 100 and 300 µm using a digital camera. The use of a digital camera in concrete image analysis has been attempted, but previous studies have required the use of a shooting cabin [15] or at least a setup in which the distance between the concrete and camera was kept constant at 300 mm [14]. In general, shotting at a specified distance is done to calibrate the area size of specimens based on the resolution or calibre of the image capturing device as well as the shooting distance [13]. However, this study provides a different method in which shooting distance can vary as long as the quality of the image is satisfactory. The dimensions of the images are instead calibrated based on the known size of mortar cube, which is 50 × 50 mm^2^. The method was prone to deviation due to angle and orientation of the image, but the deviation was tested to be minimal. In addition, this method requires significantly fewer images (18 images for every mortar mix) as compared with image analysis using machine learning or ANN [16,47] which require up to hundreds of images. However, the low number of images caused greater deviation in surface porosity between each mortar face, and this may have impacted the accuracy of the analysis.

The images were processed and analysed with Trainable Weka Segmentation and Fiji which were readily available. Fiji Particle Analyser provided the total area and provided the area of porosity for every face of the mortar cube, and then the value was adopted as a quantitative parameter in the assessment of mortar strength. In a study of mortar with pozzolanic material, the improvement of mortar strength due to the effect of pozzolanic material could be observed through a lower porosity [30]. Water absorption test and surface porosity tests conducted in this study showed a strong correlation with the 28-day compressive strength of mortar.

## 5. Conclusions

In this study, we develop a simplified image analysis methodology for the surface porosity of mortar with pozzolanic material as partial cement replacement. Type N mortar was prepared with up to 40% PSBE with an interval of 10%. A flow table test, compressive strength test, and water absorption test were conducted to obtain basic mortar properties with respect to the influence of PSBE as partial cement replacement. Images of mortar surface were taken with a digital camera. The trimmed images were processed using Trainable Weka Segmentation, and then analysed with Fiji to obtain a quantified parameter of mortar surface porosity. The flow table test showed that mortar with PSBE as cement replacement had lower workability with a greater portion of PSBE as cement replacement. The compressive strength test of mortar specimens revealed that mortar with 20% PSBE as cement replacement had the optimal strength at 28-day curing age. The improvement of strength due to lower porosity and denser microstructure was reflected in the results of the water absorption test with 20% PSBE specimens that absorbed the least amount of water. Surface porosity was quantified from an image analysis of mortar surface and also calculated that the 20% PSBE specimen had the least area of pores on it surfaces, and hence showed reliability in studying the performance of mortar with pozzolanic material. The regression analysis of PSBE and compressive strength determined a quadratic relationship among the variables. In addition, water absorption and surface porosity were strongly correlated with compressive strength. Therefore, on the basis of the results of this study, we can conclude that the simplified image analysis methodology is feasible to be explored. The primary advantages of the method are that it requires only a digital camera and has no strict limitation on shooting distance, the image processing and analysis is easy to carry out and utilises readily available programs, and it allows quantification of surface porosity as a supplementary parameter to access the performance of mortar with pozzolanic material. However, the method relies on knowing the actual size of the specimen in the images, therefore, it may be difficult to apply with large areas of concrete. In addition, it is only applicable for macro analysis of concrete. Large scale application of the method may be attempted with a higher resolution device by performing image analysis for percentage porosity while disregarding the area of a specimen. Lastly, the reliability of surface porosity can be analysed in conjunction with other parameters such as SEM and CT scanning.

## 6. Codes and Standards

1. *MS EN 197-1:2014*; Cement—Part 1: Composition, Specifications and Conformity Criteria for Common Cements. Department of Standards Malaysia: Selangor, Malaysia, 2014.
2. *ASTM C618-19*; Standard Specification for Coal Fly Ash and Raw or Calcined Natural Pozzolan for Use in Concrete. ASTM International: West Conshohocken, PA, USA, 2019.
3. *ASTM C1329-05*; Standard Specification for Mortar Cement. ASTM International: West Conshohocken, PA, USA, 2005.
4. *ASTM C1437-15*; Standard Test Method for Flow of Hydraulic Cement Mortar. ASTM International: West Conshohocken, PA, USA, 2015.
5. *BS EN 12390-3:2009*; Testing Hardened Concrete—Compressive Strength of Test Specimens. British Standards Institution: London, UK, 2011.
6. *BS 1881-122:2011*; Testing Concrete—Method for Determination of Water Absorption. British Standards Institution: London, UK, 2011.
7. *ASTM C311/C311M-18*; Standard Test Methods for Sampling and Testing Fly Ash or Natural Pozzolans for Use in Portland-Cement Concrete. ASTM International: West Conshohocken, PA, USA, 2018.

## Figures and Tables

**Table 1:** Chemical composition and physical properties of ordinary Portland cement (OPC).

Tests	Unit	Specification MS EN 197-1: 2014 CEM/B-L 32.5 N	Test Results
		Chemical composition	
Sulfate content (SO_3_)	%	Not more than 3.5	2.1
Chloride (Cl^−^)	%	Not more than 0.10	0.01
		Physical composition	
Fineness (According to Blaine)	m^2^/kg	-	440
Setting time, initial	mins	Not less than 75	155
Soundness	mm	Not more than 10	0.8
Compressive strength			
(Mortar prism), 7 days	MPa	Not less than 16	24.0
28 days	MPa	32.5 ≤ x ≤ 52.5	35.2

**Table 2:** Grading of fine aggregate.

**BS Sieve Size (mm)**	**Weight of Sieve (g)**	**Weight of Sieve + Sample (g)**	**Weight of Sample (g)**	**Cumulative %**	**Passing %**
2.360	256.60	260.90	4.30	0.85	99.15
1.180	406.30	433.90	27.60	6.29	96.71
0.600	441.80	476.90	35.10	13.21	86.88
0.425	434.10	522.20	88.10	30.58	69.42
0.300	340.70	486.00	145.30	59.22	40.78
0.150	349.10	556.00	206.90	100.00	0.00
0.000	352.40	352.20	0.00	100.00	0.00

**Table 3:** Chemical composition of PSBE and OPC.

BS Sieve Size (mm)	Weight of Sieve (g)	Weight of Sieve + Sample (g)	Weight of Sample (g)
Silicon dioxide	SiO_2_	55.82	16.05
Aluminium oxide	Al_2_O_3_	13.48	3.67
Calcium oxide	CaO	6.6	62.28
Ferrous oxide	Fe_2_O_3_	8.24	3.41
Magnesium oxide	MgO	5.94	0.56
Sodium oxide	Na_2_O	0.18	0.06
Potassium oxide	K_2_O	1.66	0.82
Phosphorus pentaoxide	P_2_O_5_	5.20	0.05
Sulpher trioxide	SO_3_	1.05	4.10
Titanium oxide	TiO_2_	1.47	0.25
Zinc oxide	ZnO	0.02	0.08
Total of SiO_2_ + Al_2_O_3_ + Fe_2_O_3_		77.54	-

**Table 4:** Flow table test results.

Mortar Specimen	Flow Table Test (%)	
	Diameter (mm)	Consistency (%)
Control	255	155
10 PSBE	250	150
20 PSBE	240	140
30 PSBE	195	95
40 PSBE	205	105

**Table 5:** Compressive strength test result.

Specimen	Compressive Strength (N/mm^2^)		
	1 Day	7 Days	28 Days
Control	2.407	9.277	10.424
10 PSBE	1.607	10.471	11.618
20 PSBE	1.016	9.436	12.254
30 PSBE	-	8.169	10.917
40 PSBE	-	4.665	7.537

**Table 6:** Image analysis output.

Slice	Count	Total Area (mm^2^)	Average Size (mm^2^)	% Area	Mean
1	326	105.308	0.323	4.104	255
2	164	57.484	0.351	2.24	255
3	196	60.257	0.307	2.348	255
4	180	59.054	0.328	2.302	255
5	215	70.854	0.33	2.761	255
6	91	13.941	0.153	0.543	255
7	174	78.859	0.453	3.073	255
8	320	86.43	0.27	3.368	255
9	265	80.942	0.305	3.155	255
10	260	109.646	0.422	4.273	255
11	232	40.376	0.174	1.574	255
12	263	67.22	0.256	2.62	255
13	210	13.313	0.063	0.519	255
14	286	108.647	0.38	4.234	255
15	243	83.386	0.343	3.25	255
16	312	82.174	0.263	3.203	255

**Table 7:** Average surface porosity of PSBE mortar.

Specimen	Average Surface Porosity (%)
Control	2.723
10 PSBE	1.664
20 PSBE	1.448
30 PSBE	2.240
40 PSBE	3.253

**Table 8:** Regression analysis of factors affecting mortar compressive strength.

Independent Variable	Dependent Variable	Correlation	R^2^	Expression
Compressive strength	PSBE %	Quadratic	0.9814	$y=-0.008x^{2}+0.253x+10.256$
	Water absorption	Quadratic	0.9021	$y=16.29x^{2}+72.63x-87.73$
	Surface porosity	Linear	0.8769	y=15.743−2.29x

## Data Availability

The data presented in this study are available on request from the corresponding author.
